# *Plasmodium relictum* infection in *Culex quinquefasciatus* (Culicidae) decreases diel flight activity but increases peak dusk flight activity

**DOI:** 10.1186/s12936-022-04265-9

**Published:** 2022-08-22

**Authors:** Dayvion R. Adams, Andrew J. Golnar, Jacob I. Meyers, Michel A. Slotman, Gabriel L. Hamer

**Affiliations:** 1 Department of Entomology, Texas A&M University, 2475 TAMU, College Station, TX 77843 USA; 2 Schubot Center for Avian Health, Department of Veterinary Pathobiology, Texas A&M University, 4467 TAMU, College Station, TX 77843 USA

## Abstract

**Background:**

Parasites are recognized for their ability to modify host physiology and behaviours in ways that increase parasite fitness. Protozoan parasites of the genus *Plasmodium* are a group of widespread vector-borne parasites of vertebrates, causing disease to a wide range of hosts, but most notably to human and avian hosts.

**Methods:**

The hypothesis that infection with the avian malaria, *Plasmodium relictum* (GRW4 lineage) impacts flight activity in one of their natural vectors, *Culex quinquefasciatus*, was tested using both parasites and mosquitoes colonized from local populations in East-Central Texas, USA. Groups of *Cx. quinquefasciatus* were allowed to feed directly on canaries with active *P. relictum* infections and control canaries with no *P. relictum* exposure history. Additionally, how *P. relictum* sporozoite invasion of mosquito salivary glands impacts mosquito flight activity behaviour was tested using a Locomotor Activity Monitor for both control and infected females. Generalized linear mixed models were used to evaluate the influence of infection status on the response variables of flight activity (continuous) and probability of flight occurring (binomial).

**Results:**

Infection status was a significant predictor of flight activity and flight probability and interactions between infection status and experimental period of infection as well as infection status and dusk were statistically significant predictors of flight activity. *Plasmodium relictum* infected mosquitoes had a mean flight activity of 3.10 and control mosquitoes had an overall mean flight activity of 3.13.

**Discussion:**

Based on these results, avian malaria parasites increase the flight activity of these mosquitoes at hours known for peak host-seeking behaviour but decrease overall diel activity.

**Conclusion:**

Although the ramifications of this behavioural change for *P. relictum* transmission are unclear, these results provide additional empirical evidence suggesting that avian malaria can influence mosquito behaviour and modulate transmission potential.

**Supplementary Information:**

The online version contains supplementary material available at 10.1186/s12936-022-04265-9.

## Background

Parasite–host co-evolution has resulted in complex adaptations, many of which lead to modifications of host behaviour or fitness. Parasites can be pathogenic to hosts, introducing adverse effects that cause morbidity or mortality [1–8]. In other relationships, parasite infection of vertebrate hosts can result in modified behaviour [9–13], or have no observable effects on host biology [14]. Vector-borne pathogens, which respond to adaptive pressure from both vertebrate and arthropod hosts, are no exception; parasite-induced alterations in vector behaviour and biology can significantly influence parasite transmission [1, 15, 16]. Vectorial capacity is a framework describing all the intrinsic and extrinsic factors that contribute to an arthropod vector’s ability to transmit pathogens, including survivorship, vector competence, and number of infective bites [17]. Parasite-induced modifications of host-seeking activity or flight activity may increase (or decrease) the number of infective bites delivered to naïve hosts or alter survivorship by decreasing the frequency of high-risk behaviours, thus changing vectorial capacity by increasing mosquito survivorship [17]. Mosquito flight is a risky behaviour that may lead to increased mortality in mosquito hosts. By modifying the flight behaviour of its mosquito host during both pre-infectious and infectious periods, *Plasmodium* parasites may increase the probability of transmission.

Although malaria parasites (*Plasmodium* spp.) are responsible for one of the most detrimental human diseases, the impact of these parasites on some aspects of their mosquito hosts remains unclear [18, 19]. Prior studies demonstrate *Plasmodium* parasites can alter mosquito survivorship, feeding behaviour, and even fecundity indicating that these parasites are generally detrimental to mosquito fitness [7, 20–25]. However, these parasite-induced modifications may also benefit the transmission of *Plasmodium* parasites. For example, Vezilier et al. [26] demonstrated that although mosquito fecundity was decreased in *Culex pipiens* mosquitoes infected with *Plasmodium relictum* (lineage SGS1), mosquito survivorship was increased. This suggests that parasites divert reproductive resources within the mosquitoes to extend their survivorship. However, other studies replicating this study found conflicting results [27], so additional studies on *Plasmodium*-mosquito interactions are needed to fully understand what occurs in nature.

Locomotion is fundamental to mosquito biology as they obtain nutrients required to survive and reproduce. Therefore, a reduction in flight in a mosquito may have negative consequences on their survival or reproductive fitness. Prior studies have identified a reduction in flight activity after infection with a pathogen. Berry et al. [28] found that the filarial nematode *Dirofilaria immitis* significantly reduced *Aedes aegypti* flight activity 8 days after infection with the parasite. Similarly, Rowland and Boersma [29] found flight activity in *Anopheles stephensi* mosquitoes was reduced by approximately 33% 17 days post-infection with *Plasmodium yoelii* parasites. A variety of parasite taxa have been shown to alter mosquito flight activity, but few studies have attempted to quantify flight activity post-infection with avian malaria lineages such as *Plasmodium relictum* [30] given the lack of in vitro culture methods and the necessity of live birds. These data enhance the ability to accurately predict disease transmission or implement vector-borne disease preventions in areas with high *Plasmodium* transmission.

The effect of *Plasmodium* parasites on the behaviour of mosquito hosts has been explored in several studies with conflicting results [19, 30, 31]. Koella et al. [21] demonstrated alterations to *Anopheles gambiae* mosquito biting behaviour that would improve the transmission of *Plasmodium falciparum* parasites, including an increase in multiple biting events. Other studies have shown that vertebrate hosts infected with *Plasmodium* spp. are more attractive to both infected and uninfected mosquitoes, suggesting that the parasites are altering the host-seeking behaviour of the mosquitoes [23, 25, 32]. However, other studies have demonstrated no effect on the attractiveness of mosquitoes to *Plasmodium*-infected vertebrate hosts, or that it is dependent on the intensity of infection [33, 34]. Some of this can be explained by biological factors, but differences in experimental designs can also influence observations [30]. Nonetheless, modifications to mosquito-host attraction by *Plasmodium* parasites can have important ramifications for transmission and warrants further study.

Avian *Plasmodium* spp. (avian malaria) provide a model system to study the effects of parasite infection on the behaviour of their mosquito hosts [35]. Avian malaria lineages are closely related to human *Plasmodium* parasites and have a cosmopolitan distribution [36, 37]. Because of this, many studies utilize avian malaria parasites to study vector-parasite interactions to contrast relationships in multiple systems. Here the flight activity of *Culex quinquefasciatus* mosquitoes infected with the avian parasite *Plasmodium relictum* (GRW4 lineage) was quantified. Alteration of the diel flight activity of *Cx. quinquefasciatus* mosquitoes at different phases of the parasite development is hypothesized to be the result of this study.

## Methods

### Bird and Plasmodium collection

Wild house sparrows (*Passer domesticus*) were captured during the summer of 2020 in College Station, Texas using 12-m mist nets with 30 mm mesh size (Association of Field Ornithologists, Portland, ME). Each bird was immediately exsanguinated via jugular venipuncture to recover as much blood as possible (up to 1000 µl usually) for parasite inoculation and molecular diagnostics. Exsanguination was carried out using a 28 ½ gauge syringe that was treated with heparin (Sagent Pharmaceuticals, Schaumberg, IL, USA) to prevent clotting. About 1–2 µl of blood was used to create two thin blood smears that were air dried, fixed in 100% methanol, and later stained with Giemsa [36]. The remaining blood was kept on ice and stored up to 2 days at 4 °C until use. The stained smears were screened in 10 × 10 fields of view at 40× magnification to determine the presence of *Plasmodium* as well as any other haemosporidians and trypanosomes, with each field of view having approximately 1000 red blood cells. Screening of blood smears was done twice from different fields of view to ensure no false negatives. Only one house sparrow had an active infection with *P. relictum* that was utilized for future inoculations. The blood from the *P. relictum* infected house sparrow tested negative for diverse arboviruses by RT-PCR, per institutional requirements associated with biosafety. All work with wild birds was approved by the Texas A&M University Institutional Animal Care and Use Committee (IACUC AUP 2018-0144) and Texas Parks and Wildlife Scientific Research Permit (No. SPR-0512-917).

### Mosquito colony and maintenance

*Culex quinquefasciatus* colonized in the summer of 2018 from College Station, Texas [5] were utilized for these experiments, and were between F20–25 generations removed from the wild. Mosquitoes were maintained on a natural night and day light cycle (10.5 h light, 14.5 h dark) with a constant 50% humidity at 27 °C. Adult mosquitoes in the colony were maintained on commercially acquired chicken blood treated with Alsever’s solution (Hemostat, Dixon, CA) and a 10% sucrose solution was provided ad libitum.

### *Plasmodium relictum* propagation

*Plasmodium relictum* (GRW4 lineage) obtained from wild house sparrow blood (50–100 µl) was initially inoculated into two domestic canaries (*Serinus canaria*). It was then passaged two additional times until a high parasitaemia of 10% of red blood cells was observed. This was done since initial inoculations of *P. relictum* were low parasitaemia and difficult to detect via microscopy. *Plasmodium* used for the current study was passaged between 3 canaries from the wild sparrow before exposure to mosquitoes. Passages occurred on Day 7 post-inoculation, which corresponded to the peak parasitaemia in utilized canaries. Jugular venipuncture was the route of inoculation for all canaries and was allowed to amplify in canaries for up to 10 days post-inoculation. Infections were monitored and confirmed using microscopy and PCR [36, 38]. Methodology for microscopy on canary infections is the same protocol as above. Canaries were routinely screened via microscopy on days 4 and 7. If canaries had not cleared the infection by day 10, canaries were exsanguinated, and blood was cryopreserved for future study. Canaries that cleared infection were euthanized and were not utilized again for passaging. All work with captive canaries was approved by Texas A&M University Institutional Animal Care and Use Committee (AUP 2018-0175).

### Mosquito infection

**Fig. 1:**
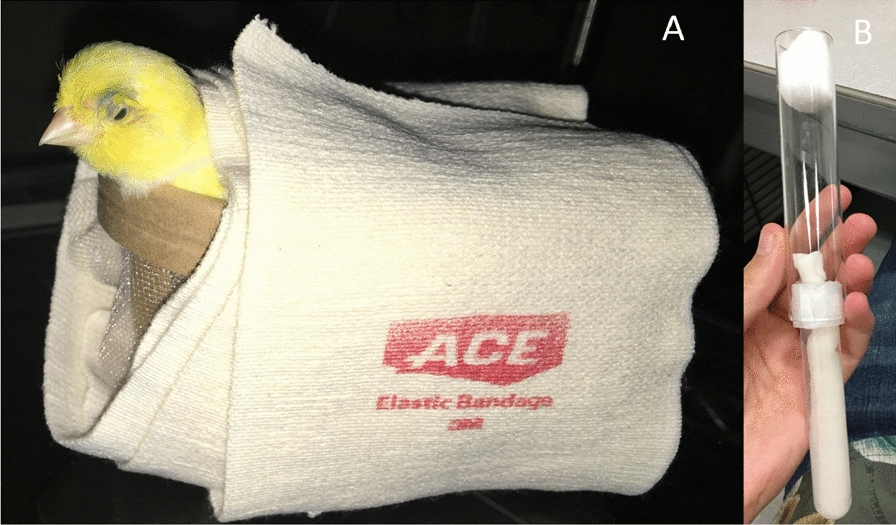
**A** Restrained blood feeding of female *Culex quinquefasciatus* on a domestic canary. Athletic bandage was used as a restraint. Canaries were restrained for up to 30 min. **B** Mosquitoes were kept alive for 10 days in a glass tube placed into the *Drosophila* Locomotor Activity Monitor 25. On one end, plastic test-tubes were used to hold the 10% sucrose solution and a sugar wick. The tubes were held in place with multiple layers of parafilm for a tight fit. On the other end, a cotton ball was used to keep the mosquito within the glass tube. Careful attention was paid to prevent the wick and cotton ball from extending too far into the glass tube so that erroneous recordings were not collected

Four trials (replicates) of mosquito infection were conducted given the constraint on the number of individual mosquitoes that could be monitored for flight activity at a given time. In each trial, cohorts of 100 one week old female *Cx. quinquefasciatus* were offered either a canary between 6 and 8 days post-infection with *P. relictum* (GRW4 lineage) with a parasitaemia of about 10% or a control canary that had never been exposed to *Plasmodium* (verified using PCR and microscopy). Feeding events occurred between 5–7 am as prior work with these colonized *Cx. quinquefasciatus* determined that this period yields the highest blood feeding success. To do this, canaries were restrained with flexible athletic bandage over a container with mesh for mosquito feeding on the feet and legs of the canary for up to 30 min (Fig. 1A).

Mosquitoes were then knocked down on ice, and only fully engorged females were removed and added to a separate container (plastic 16 cm by 21 cm cup with mesh lid) with other engorged females, which were given a Sella score to quantify the blood meal size [39]. The blood engorged mosquitoes were provided 10% sucrose solution (ad libitum) and allowed to develop eggs. A small cup containing water was placed into the cage on day 6 post-infection and retrieved on day 8 post-infection. On day 8 post-infection, mosquitoes were knocked down and 16 of those mosquitoes that had laid eggs were individually removed and placed into glass tubes for activity monitoring in the *Drosophila* Locomotor Activity Monitor 25 (LAM 25) (TriKinetics Inc, Waltham, MA). Each tube had a cotton ball on one end to prevent escape, and a 10% sucrose solution on the other (Fig. 1B). The activity monitor houses 32 mosquitoes at once (8 columns by 4 rows), so 16 mosquitoes exposed to *P. relictum* and 16 mosquitoes fed on control birds were simultaneously examined. Treatments were spaced so that they would occupy every other row (i.e. 8 infected, 8 controls). Mosquitoes were then left in the activity monitor inside an environmental chamber for 10 days undisturbed at 56% humidity, 27 °C, and a light-dark cycle of 12 h light followed by 12 h of dark (19.3 cu ft. Peltier Refrigerated Incubator, Shel Lab, Cornelius, OR). On day 18 post infection, mosquitoes were removed, and *Plasmodium*-exposed individuals had midguts and salivary glands dissected for confirmation of infection. Midguts were stained with 0.05% mercurochrome solution for identification of oocysts at low magnifications, and subsequently preserved for DNA extraction of oocysts and midguts [36]. Salivary glands were added to a microscope slide containing a drop of phosphate buffer solution, erupted using dissecting probes, and allowed to dry [36]. Using the same method as blood smears, salivary glands were stained and sporozoites were identified under 100× oil immersion. Heads and thoraces were preserved for DNA extraction of sporozoites.

### Molecular diagnostics and sequence determination

DNA from infected mosquito head/thorax and midgut tissue was extracted to detect the presence of sporozoites and oocysts, respectively. This was done following the Bio-tek E.Z.N.A. (Omega, Norwalk, CT) manufacturer recommendations for DNA extraction (tissue) with slight modification; samples incubated at 70 °C for a minimum of 1 h. Polymerase Chain Reaction (PCR) was run on all samples to confirm infection in thoraxes and midguts using HAEMF and HAEMR2 primers by amplifying a 478 bp region of the cytochrome b gene following previously published thermocycling parameters [38]. The same method was used for identification of parasite in bird blood, except DNA from blood was extracted using the Bio-tek E.Z.N.A. blood extraction protocol. Parasite lineage determination was done by Sanger sequencing in forward and reverse directions (Eton Biosciences Inc., San Diego, CA). Clean sequences were queried using GenBank and MalAvi databases to identify similar sequence matches.

### Data analysis

**Fig. 2:**
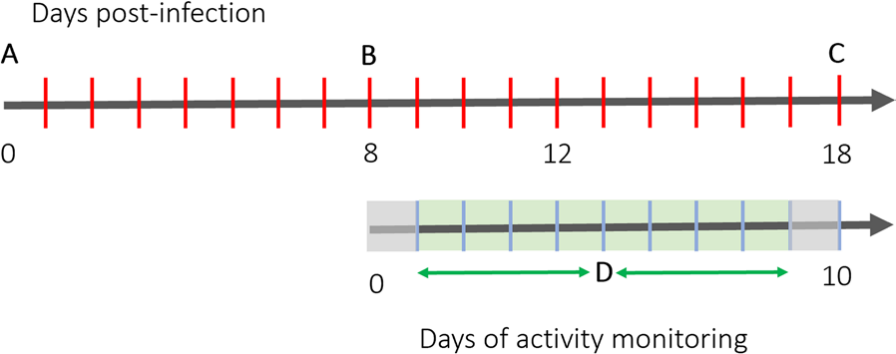
An experimental timeline of activities during experimentation. The top line refers to the post-infection days of *Cx. quinquefasciatus* with *P. relictum* (18 days total). The bottom line refers to the days that mosquito activity was being recorded in the environmental chamber (10 days total). Gray shaded days were removed from analyses, and green shaded days were included. Letters represent points of interest as follows. **A** Mosquitoes were infected with *P. relictum*, **B** mosquitoes that laid eggs were added to the activity monitor, **C** all mosquitoes were removed from the activity monitor and dissected, **D** the midpoint separating early and late day analyses relating to the timing of sporozoite entry into the mosquito salivary glands. Day 12 post-infection corresponds with when it is expected to see sporozoite presence in mosquito salivary glands

Mosquito activity was only analysed for days 9 through 16 post-infection of activity monitoring in the environmental chamber (Fig. 2) for acclimation. Mosquito activity was recorded at 30 s intervals and combined into 30-min increments for analysis consistent with prior research utilizing this locomotor activity monitor [40, 41]. In this study, data from females that were confirmed to be infected with *Plasmodium*, either molecularly or by sporozoite microscopy post activity monitoring were analysed. The uninfected control mosquitoes included only those that fed on the uninfected canary. Any mosquitoes (infected or control) that died during flight activity recording at any time during the 8 days were removed from analysis.

A generalized linear mixed model (GLMM) was run to identify the best fit for continuous activity data using the zero inflated negative binomial family due to the frequency of no flight events zero-inflated data [42]. For this model, the response variable was the number of beam-breaks in each 30-min bin across the 8 days. Fixed variables were infection status, period of observation (e.g. early vs. late), dawn, dusk, non-peak activity time, as well as a three-way interaction between infection status, period of observation, and dusk and their corresponding two-way interactions (Table 1). Individual mosquitoes nested within trial was included as the random effect for these models. A stepwise removal of terms was done until a best fit model was obtained. All models were compared using Akaike information criteria (corrected, AICc), and the model with the lowest AICc was selected as the best fit model. Additional generalized linear mixed models (GLMM) were run to identify the best fit for the probability of a flight event during a 30 min bin across 8 days using the binomial family. For these models, the response variable is a binomial of two possibilities: flight observed or not. Fixed and random effects were the same as above, and model selection was also the same. Additional file 1: Tables S1 and S2 provide specific model effect terms and their corresponding AICc values.

**Table 1:** Variable statistics for the best fit negative binomial GLMM with the response variable as continuous *Cx. quinquefasciatus* flight activity across by 30 min bins across the 8 days of study

Variable	Estimate	SE	Z value	p-value
Intercept	– 1.046	0.306	– 3.417	< 0.001
Infection status (Control)	**0.563**	**0.270**	**2.082**	**0.038**
Dusk (True)	**3.880**	**0.225**	**17.252**	**< 0.001**
Dawn (True)	**2.296**	**0.119**	**19.350**	**< 0.001**
Period of observation (Late)	**0.917**	**0.110**	**8.359**	**< 0.001**
Nonpeak (True)	**– 0.290**	**0.084**	**– 3.433**	**< 0.001**
Infection status (Control) * Dusk (True)	**– 0.754**	**0.296**	**– 2.545**	**0.011**
Infection status (Control) * Period (Late)	**– 0.261**	**0.141**	**– 1.858**	**0.063**
Dusk (True) * Period (Late)	**– 0.631**	**0.321**	**– 1.964**	**0.049**
Infection status (Control) * Dusk (True) * Period (Late)	– 0.060	0.422	0.142	0.887

Variables in bold represent statistically significant variables within the model. Asterisks represent an interaction within the model. Words in parentheses are the reference variable

**Fig. 3:**
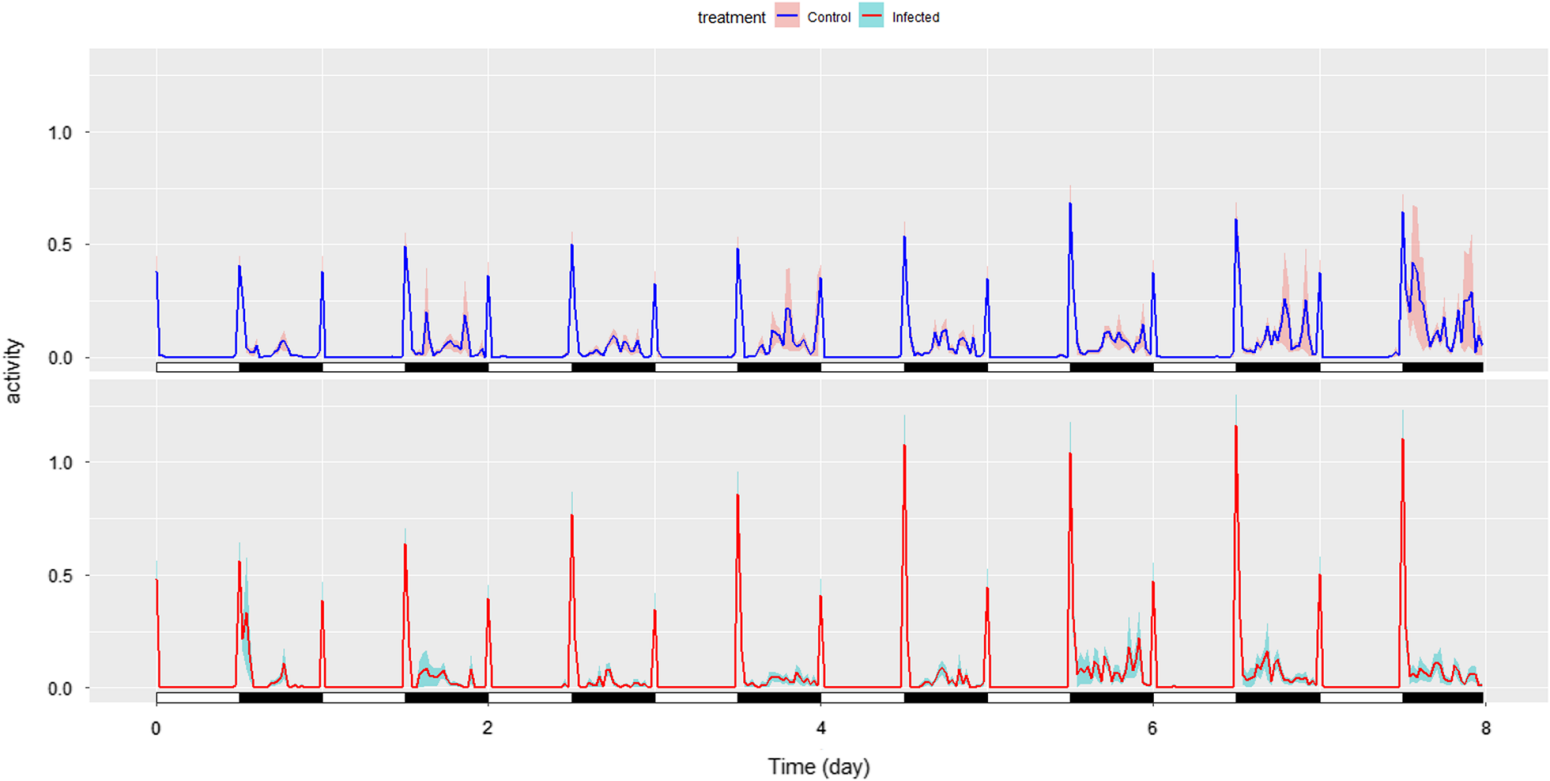
The actogram of *P. relictum* infected and uninfected control *Culex quinquefasciatus* mosquitoes’ activity at specific time points across the 8 days of observation. Black and white bars represent scotophase and photophase, respectively, and combined, they represent a single day. Activity is represented by average activity of all mosquitoes in that treatment at the time point during a particular time interval binned by 30-min increments with 95% confidence intervals depicted as lightly shaded colours. 47 mosquitoes were analysed for infected mosquitoes and 47 mosquitoes were analysed for control mosquitoes

Zeitgeber time (ZT) is frequently utilized in circadian rhythm analysis as a description of when lights are turning on and off. Here ZT 0 represents 7:30 am when lights turn on (dawn), and ZT 12 represents 7:30 pm when the lights turn off (dusk) (Fig. 3). The mosquito actogram was created using the Rethomics framework [43]. Analyses were performed using the R statistical software v3.5.2 (R Foundation for Statistical Computing, Vienna, Austria). Negative binomial GLMM was done in glmmTMB package [42] and binomial GLMM was done in lme4 package [44]. Means are presented ± standard error, and an alpha value of p = 0.05 was used for judging statistical significance.

## Results

### The collection of *Plasmodium* infected birds

Twenty-one house sparrows were exsanguinated on June 15, 2020 in College Station, Texas, USA. Only one of the birds (male) tested positive for Haemosporida infection via microscopy and PCR. After sequencing and consensus sequence was generated, the closest match in the NCBI BLAST database was a 99.58% match to sequence MG018687.1 which is *Plasmodium relictum* (GRW4).

### Infected vs. control flight activity comparison

When comparing mean total flight activity (i.e. 24 h across 8 days), a slight difference in mean total diel flight activity was observed between the control and *Plasmodium*-infected mosquitoes. Control mosquitoes had a mean total flight activity of 3.13 ± 0.15 beam breaks throughout the 8 days of observation, and infected mosquitoes had a mean total flight activity of 3.10 ± 0.13 beam breaks. The negative binomial GLMM analysis for continuous flight activity best fit model included all fixed variables as well as an interaction variable between infection status, period of observation, and dusk. All terms except the interaction between infection status/period/dusk were significant predictors of model fit (Table 1). Even though this term was not significant, it improved the overall fit (i.e. lowest AICc value) and was included in the final model. Given the significance of the two-way interaction terms, their effects on flight activity and not their first-order main effects were considered. The binomial GLMM analysis for flight activity probability best fit model included all original fixed variables except peak activity (Table 2). In the best fit binomial model, all variables were significant except interactions between infection status/dusk, infection status/period of observation, and infection status/dusk/period of observation (Table 2). Given the significant result the dusk:period interaction, only the effect of the interaction on the probability of flight occurring and not its first-order main effects were considered.

**Table 2:** Variable statistics for the best fit binomial GLMM with the response variable as *Cx. quinquefasciatus* mosquitoes did or did not take flight during by 30 min bins across the 8 days of study

Variable	Estimate	SE	Z value	p-value
Intercept	– 3.046	0.185	– 16.447	< 0.001
Infection status (Control)	**0.318**	**0.145**	**2.177**	**0.030**
Dusk (True)	**2.616**	**0.097**	**26.928**	**< 0.001**
Dawn (True)	**0.460**	**0.073**	**6.293**	**< 0.001**
Period of observation (Late)	**1.090**	**0.055**	**19.831**	**< 0.001**
Infection status (Control) * Dusk (True)	– 0.080	0.129	– 0.616	0.538
Infection status (Control) * Period (Late)	0.009	0.090	– 0.095	0.924
Dusk (True) * Period (Late)	**– 0.369**	**0.144**	**– 2.562**	**0.010**
Infection Status (Control) * Dusk (True) * Period (Late)	– 0.053	0.187	– 0.284	0.776

Variables in bold represent statistically significant variables within the model. Asterisks represent an interaction within the model. Words in parentheses are the specific variable type (i.e. Infected or Control)

## Discussion

The ability of avian malaria lineage *P. relictum* (GRW4) to modify the flight activity of an avian malaria vector, *Cx. quinquefasciatus*, as well as the probability of flight occurring for infected and control mosquitoes was evaluated. These results demonstrate that *P. relictum* infection in *Cx. quinquefasciatus* mosquitoes decreases overall diel flight activity of infected mosquitoes, but increased activity during crepuscular intervals (Fig. 3). The binomial model suggests that there is a significant effect of infection status on the probability of flight occurring, with control mosquitoes more likely to fly at any given time and had more dispersed activity (Table 1). The dawn variable is expected to be significant given the increased flight activity at this crepuscular time (Fig. 3). In this model, mosquitoes were less likely to fly when considering an interaction between dusk and the latter half of the experiment, which is surprising considering infected mosquitoes expressed increased flight activity during both time periods, but this interaction is considering all mosquito activity combined (control and infected).

In this experiment, all included variables were statistically significant predictors of continuous mosquito flight activity except the three-term interaction variable (Table 1). While some of these are to be expected (i.e. mosquitoes fly more at dawn than in non-peak times), the interaction variables of this model provides an explanation for observations in the actogram. *Culex* mosquitoes are known to be crepuscular in host-seeking behaviour [45], and so peaks during these times were expected. The increase in flight activity around dusk may translate to increased host-seeking activity and contact with birds, which could increase transmission of *Plasmodium*. The effect of the interaction variable for infection status and dusk (ZT 12–14) time periods on continuous flight activity suggests that control mosquitoes flew less than *P. relictum* infected mosquitoes during scotophase transition hours (dusk). This explains the increased activity of infected mosquitoes during this time on the actogram when compared to control mosquitoes (Fig. 3) and could be due to more resources diverted to host-seeking, which would increase the probability of successful parasite transmission to additional vertebrate hosts. Finally, the interaction term between infection status and late day infection means that control mosquitoes flew significantly less than infected mosquitoes after day 12 post-infection. A possible cause is the presence of sporozoites within infected mosquitoes causing them to host-seek more due to parasite manipulation. These sporozoites would be less present in earlier stages of the experiment when it is expected that *P. relicitum* oocysts are present within the mosquito midgut.

Other studies have demonstrated alterations to mosquito host-seeking and feeding behaviour including increases in host-attractiveness to *Plasmodium*-infected mosquitoes and multiple biting events [21, 23, 25, 32]. Alternatively, other studies have demonstrated no effect of *Plasmodium* on the flight activity of their mosquito hosts [46]. The effect of *Plasmodium* on mosquitoes in these studies may change depending on the mosquito species studied, *Plasmodium* species utilized, as well as natural or unnatural systems. This study utilized a *P. relictum* lineage that was collected locally from wild birds as well as a *Cx. quinquefasciatus* colony that was recently established from local populations to emulate what is observed in nature. Similar studies have evaluated infection of mosquitoes in different pathogen systems and have also identified modifications to flight activity. This means that parasite alteration of mosquito flight activity behaviour is not isolated to *Plasmodium* mosquitoes and may be widespread across parasitism [13, 28, 29].

The flight activity of *Cx. quinquefasciatus* mosquitoes was quantified in an activity monitor that detects beam breaks for every flight event as a proxy for host-seeking behaviour, but this approach has limitations. Flight activity at any given time can contribute to multiple behaviours including host-seeking, oviposition seeking, or sugar source-seeking. To eliminate one of these behaviours, only mosquitoes that laid eggs prior to the quantification of flight activity were utilized for experiments. However, this does not explicitly mean the mosquitoes were host-seeking. Models from these experiments indicate a decrease in the total diel activity of mosquitoes with *Plasmodium* infection which could be the result of diverted resources within the mosquito. However, *Plasmodium* infection significantly increased flight activity shortly following the light to dark transition which would increase energy expenditures during this crepuscular period, potentially because of *P. relictum* manipulation. Other studies have also demonstrated *Plasmodium* parasites diverting resources to facilitate further transmission of the parasites. Vézilier et al. [26] also found that *Cx. quinquefasciatus* mosquitoes infected with *Plasmodium relictum* (SGS1 lineage) had increased survivorship, but decreased fecundity, which could serve the purpose of furthering the parasite transmission. A decrease in overall *Cx. quinquefasciatus* flight activity post-infection benefits the *P. relictum* pathogen as it can complete sporozoite development and invasion of the salivary glands and potentially infect another avian host.

In this study, an increase in activity between early and late *P. relictum* infected mosquitoes was quantified. Day 12 post-infection is generally accepted as within the range of time (7–16 days post-infection) where *P. relictum* oocysts rupture, and sporozoites travel to the mosquito salivary glands [47, 48]. On day 5 of the analysis, an overall increase in flight activity (total and peak) (Fig. 3) was observed, which is consistent with higher sporozoite infection levels in mosquitoes. This behavioural pattern may be suggestive of parasite manipulation as overall decreased flight activity may optimize *Plasmodium* transmission by permitting mosquitoes to develop sporozoite infections in the salivary glands before partaking in crepuscular host-seeking behaviour. Reduced flight activity during the day might limit risk of death and permit host-seeking during dusk when probability of obtaining a blood meal from and avian host could be higher.

Alternatively, behaviours identified here may, in part, be due to alterations in mosquito tolerance to light changes. Multiple studies have removed the first two hours of mosquito activity data after light transitions due to the “startle response” that occurs when mosquitoes experience light change in artificial settings [12]. This response to the changing light was also observed in the current study, although from field studies *Cx. quinquefasciatus* is also considered crepuscular [49]. This response to changing light might have increased activity via a startled response, but *P. relictum*-infected individuals had higher activity during these transition times, suggesting some effect on flight behaviour by infection (Fig. 3).

The measure of flight activity recorded with the instrument in this study is not a direct measure of host-seeking behaviour. Prior studies in the avian malaria system have found that *P. relictum* infected and uninfected mosquitoes were highly attracted to *P. relictum* infected birds, supporting the claim that mosquito host-seeking behaviour is altered by this parasite [23, 32, 34]. Additionally, a study by Lacroix et al. [22] in the human malaria system found similar results. However, other studies have found that there was no evidence of this alteration, and perhaps a parasite avoidance by these mosquitoes [31, 50]. These data support a modulation in flight activity by *P. relictum* in less overall flight activity and increased flight activity at peak host-seeking times, which could lead to an increase the transmission of this *P. relictum* parasite. However, the sample size is relatively low given the constraints of the experiment including the acquisition of infection by mosquitoes and canaries and an instrument that can only record flight activity for 32 individuals at a time. The observations of this study warrant further host choice experiments to see if *P. relictum*-infected *Culex* mosquitoes increase contact and feeding on birds in a controlled setting as well as follow up studies with larger sample sizes to see if the same effect of infection on flight activity is observed.

Parasitaemia within vertebrate hosts and mosquitoes are important factors that may influence the intensity of flight activity modulation. This study utilized canaries that had active infections with a parasitaemia of about 10% which is extremely high and would not usually be observed in a natural setting, with natural infections ranging between 0.5 and 6% depending on the environment and avian species sampled [35, 47, 51]. Because of this, the effect that was observed in mosquitoes may be exaggerated. Mosquito parasitaemia is also an important factor that may explain variation in infected mosquito flight activity. Prior studies have observed an enhanced effect of *Plasmodium* infection on various biological factors including fecundity and survivorship, and so that should be considered when conducting experiments like these [16, 33].

While mosquitoes were screened for the presence or absence of sporozoites and oocysts, parasitaemia within mosquitoes was not quantified. Mosquito parasitaemia could have enhanced the effect of infection on mosquito flight activity and should be considered when interpreting the results of this study. Future studies should consider parasitaemia of individual mosquitoes as a variable to quantify the effect of parasitaemia on flight activity of mosquitoes.

While mosquito infection with *P. relictum* is one explanation for modulations in flight activity, there are other possible explanations for these changes. Mosquito blood meal quality is an important factor that determines how nutritive blood is to a mosquito and influences various factors, such as fecundity and mortality. Infected birds have lower quality of blood when infected with *P. relictum* due to depleted red blood cells and increased immunocytes [36]. Because of this, infected mosquitoes may have obtained overall less nutrition after digesting blood meals, leading to decreased flight activity. This could explain the overall decreased activity in infected mosquitoes, but not the increased crepuscular activity that was observed.

Overall, our study observes an effect of *P. relictum* infection on the flight activity of *Cx. quinquefasciatus* mosquitoes during crepuscular hours. To the author’s knowledge this is the first study to quantitatively evaluate the effect of avian malaria on *Culex* mosquito flight behaviour. This was done this using a natural system combining *P. relictum* (GRW4) and its vector, *Cx. quinquefasciatus*, colonized from the same study location of College Station, Texas [5]. Although these results provide further evidence that *Plasmodium* infection can modify vector behaviour, further work is needed to determine if increases in flight activity during peak hours of host-feeding promote parasite transmission. Future studies should seek to understand why *P. relictum* decreases the total flight activity of *Cx. quinquefasciatus* mosquitoes, and how these behavioural modifications might impact vector-control strategies.

## Supplementary Information

**Additional file 1.** Mixed effect model selection reporting AICc values when evaluating the influence of *Plasmodium relictum* infection on *Culex quinquefasciatus* flight. **Table S1.** Mixed effect model selection reporting AICc values when evaluating the influence of *Plasmodium relictum* infection on *Culex quinquefasciatus* probability of flight using the binomial response variable. **Table S2.** Mixed effect model selection reporting AICc values when evaluating the influence of *Plasmodium relictum* infection on *Culex quinquefasciatus* continuous flight activity.

## Data Availability

The datasets supporting the conclusions of this article are available in the Texas A&M Oaktrust repository, https://hdl.handle.net/1969.1/19585.
