# Supplementary material for: Plasmodium relictum infection in Culex quinquefasciatus (Culicidae) decreases diel flight activity but increases peak dusk flight activity
Source: Malar J. 2022 Aug 22;21:244. doi: 10.1186/s12936-022-04265-9 (PMC9396771; doi:10.1186/s12936-022-04265-9)
Supplement: Supplementary file 1 — Additional file 1. Mixed effect model selection reporting AICc values when evaluating the influence of Plasmodium relictum infection on Culex quinquefasciatus flight. Table S1. Mixed effect model selection reporting AICc values when evaluating the influence of Plasmodium relictum infection on Culex quinquefasciatus probability of flight using the binomial response variable. Table S2. Mixed effect model selection reporting AICc values when evaluating the influence of Plasmodium relictum infection on Culex quinquefasciatus continuous flight activity. [file 12936_2022_4265_MOESM1_ESM.docx]

**Table S1**.

| **Fixed Effects** | **AICc** |
| --- | --- |
| Binomial ~ Infection Status + Period + Dawn + Dusk + Non-Peak | 20364.9 |
| Binomial ~ Infection Status + Period + Dawn + Dusk | 20363.2 |
| Binomial ~ Infection Status * Period + Dawn + Dusk + Non-Peak | 20366.9 |
| Binomial ~ Infection Status * Period + Dawn + Dusk | 20363.2 |
| Binomial ~ Infection Status * Period * Dusk + Dawn + Non-Peak | 20351.5 |
| Binomial ~ Infection Status * Period * Dusk | 20350.1 |

* indicate an interaction in the model. All models included individual mosquitoes nested within trial as random effects.

**Table S2**.

| **Fixed Effects** | **AICc** |
| --- | --- |
| Flight Activity ~ Infection Status + Period + Dawn + Dusk + Non-Peak | 54837.8 |
| Flight Activity ~ Period + Dawn + Dusk + Non-Peak | 54837.7 |
| Flight Activity ~ Infection Status * Period + Dawn + Dusk + Non-Peak | 54837.3 |
| Flight Activity ~ Infection Status * Dusk + Period + Dawn + Non-Peak | 54828.8 |
| Flight Activity ~ Infection Status * Period * Dusk + Dawn + Non-Peak | 54823.2 |

* indicates an interaction in the model. All models included individual mosquitoes nested within trial as random effects.
